# Risk of Increasing the Sudden Diagnosis of AIDS in Japan during and after the COVID-19 Outbreak

**DOI:** 10.31662/jmaj.2021-0174

**Published:** 2021-12-24

**Authors:** Masamine Jimba, Motofumi Suzuki, Tadaichi Kitamura, Rogie Royce Carandang, Nancy L Sieber

**Affiliations:** 1 Department of Community and Global Health, Graduate School of Medicine, The University of Tokyo, Tokyo, Japan; 2 Department of Urology, Tokyo Metropolitan Bokutoh Hospital, Tokyo, Japan; 3 Department of Urology, Graduate School of Medicine, The University of Tokyo, Tokyo, Japan; 4 Harvard T.H. Chan School of Public Health, Harvard University, Boston, USA

**Keywords:** HIV, AIDS, COVID-19, health service, Japan

## Abstract

Public health centers have played an important role in controlling the spread of COVID-19 in Japan. However, the staff members of 469 centers have been overwhelmed by the huge increase in workload, and some public health centers were obliged to temporarily stop regular HIV testing. With the halting of HIV testing during the COVID-19 crisis, the proportion of “*Ikinari*-AIDS” or a sudden diagnosis of AIDS without prior knowledge of the HIV infection status is expected to rise. To provide essential public health services, it is time for Japan to focus on delivering public health services beyond the existing public health centers.

## Introduction

Japan has been successful in controlling the coronavirus disease (COVID-19), with relatively low morbidity and mortality compared with some countries in Europe, Asia, and the Americas. The total number of reported deaths was just 16,313 as of September 9, 2021 ^(1)^. Among other factors, the 469 public health centers located throughout Japan have played an important role in controlling the spread of COVID-19.

However, the impact of COVID-19 is not limited to the morbidity and mortality it causes directly. It also has a detrimental effect on the delivery of routine, essential health services. In March 2020, WHO issued a key document warning about the challenge of maintaining basic health services during the pandemic. Countries have to make tough decisions to balance the demands of the COVID-19 response while engaging in strategic planning and coordinated action simultaneously. As anticipated, the public health system in Japan has indeed been overwhelmed by the demand for PCR testing and the need to respond to positive and negative cases.

Because of the high demand for COVID-19 control, many routine and elective services have been postponed or suspended in Japan. For instance, public health centers’ Human Immunodeficiency Virus (HIV) testing has been cancelled indefinitely during the COVID-19 outbreak ^(2)^. This cancellation may have led to public health concerns related to HIV/AIDS epidemiology in Japan during the pandemic. For example, Japan’s current HIV testing system seems to have missed more HIV cases without an Acquired Immunodeficiency Syndrome (AIDS) diagnosis during the pandemic ^(3)^. While Ejima et al. presented the proportion of HIV cases with an AIDS diagnosis increased significantly in the second quarter of 2020, they did not differentiate “late testers” and those with “sudden” cases of AIDS in their analysis ^(3)^. This article describes the concern that Japan is at risk of an increase in “sudden” cases of AIDS in the context of COVID-19 and proposes a way forward.

## Challenges Faced by the Public Health Centers in the Context of COVID-19

In Japan, public health centers carry out a wide range of duties. Their major work is preventing infectious diseases, collecting vital statistics data, and providing other community-level public health services ^(4)^. On January 14, 2020, Japan reported its first case of COVID-19, an imported case. Japanese authorities expanded the state of emergency into all of Japan on April 16, 2020. Throughout the ongoing outbreak, workers of public health centers have focused their efforts on the surveillance of COVID-19 cases, answering phone calls from returnees and close contacts, and transporting people to hospitals.

To meet the demands of the COVID-19 crisis, the public health centers have had to stop testing for routine infectious diseases. For example, the public health center in *Shinjuku-ku*, one of the largest cities in Tokyo, announced the temporary cancellation of HIV testing and testing for other Sexually Transmitted Infections (STIs), including syphilis, chlamydia, and hepatitis B. The date when testing will resume has not been determined ^(2)^.

New cases of HIV/AIDS have exceeded 1,300 per year since 2006 in Japan. In 2018, 940 new “HIV cases” (without AIDS symptoms) and 377 new “AIDS cases” were detected ^(5)^, which suggests that approximately one-third of HIV/AIDS cases are not diagnosed with HIV until the patients seek care for AIDS symptoms. Such patients are considered to have “*Ikinari*-AIDS,” a term coined in Japanese. “*Ikinari*” is a Japanese word meaning an “unexpected, sudden” situation, and “*Ikinari-AIDS”* refers to a situation when HIV-positive people are diagnosed with AIDS without knowing their HIV infection status.

## Risk of Increasing “*Ikinari-AIDS*” during the COVID-19 Crisis

With the halting of HIV testing during the COVID-19 crisis, the proportion of “*Ikinari*-AIDS” (denominator is HIV/AIDS cases) is expected to rise. Anonymous testing for HIV and other STIs is normally available for free at public health centers in Japan ^(5)^, making it easy for high-risk populations to be regularly tested for HIV, even in the absence of symptoms. We expect that ending HIV testing in public health centers may decrease the number of tests performed on members of the targeted high-risk populations. Ejima et al. supported our assumption as they stated a 73% reduction in the number of tests conducted in the public health centers and municipalities in Japan compared with the year before the COVID-19 pandemic ^(3)^. This reduction in testing may imply that we could not fully capture the epidemiological situation of HIV/AIDS in Japan during the COVID-19 pandemic. It is more likely that there is a substantial proportion of undiagnosed cases, and whether the incidence increased (or decreased) during the COVID-19 pandemic is presently unknown.

Early detection of the HIV status reduces transmission because it leads to changes in risky behaviors and the reduction of viral load in patients receiving medical intervention. Late diagnosis of HIV is associated with lower Cluster of Differentiation-4 (CD4) counts and the need for more expensive HIV-related hospital care and anti-retroviral drugs. Furthermore, medical costs for AIDS-naive HIV patients are reported to be about one-fourth of what they are after the onset of AIDS.

## The Way Forward

The term “*Ikinari*-AIDS” is similar to the term “late testers” of the HIV status, which has been reported in the US, France, and Thailand. The Center for Disease Control and Prevention (CDC) in the US defines “late testers” as persons who had their first positive HIV test less than one year before the diagnosis of AIDS. They noted that 65% of late testers sought HIV testing because they became aware of symptoms, and 87% of late testers had their first positive HIV test at an acute or referral medical care setting. While there are some differences in the timing of their initial diagnosis, both “*Ikinari-AIDS*” patients and “late testers” share a common characteristic: they do not come to health facilities to check their HIV status in the early stages of their infection.

What, then, can we do? We recommend that populations at high risk of STIs seek their routine HIV testing in medical facilities such as clinics and hospitals, particularly facilities with expertise in STIs, rather than public health centers. Although a self-testing kit for HIV is available in Japan, Japanese health authorities have not approved it due to technical issues ^(3)^. Moreover, healthcare providers should be aware of our recommendation for HIV testing, as well as current HIV epidemiology trends to promote HIV testing. That is why we should determine if the rate of “*Ikinari*-AIDS” in Japan is increasing in the context of COVID-19.

However, recommending behavioral changes among high-risk populations and healthcare providers is not enough. For example, all should be aware that HIV testing in medical facilities is not always covered by Japanese medical insurance. The COVID-19 crisis has changed the landscape for community-level public health services in Japan for the foreseeable future.

The crisis, however, creates an opportunity to reform the operation of public health centers to make them better able to carry out their missions in the face of a surge in demand. It is a chance to determine which are the “essential health services,” including HIV testing, and to reduce the burden of “non-essential health services.” The US CDC recommends that public health strategists re-assign staff members from less busy services to assist with essential services and that they work with the Ministry of Health and local healthcare workers’ societies to determine how task shifting can best be used to provide essential services. This advice is relevant in Japan, and the government should focus on delivering public health services beyond public health centers to provide truly essential health services.

## Article Information

### Conflicts of Interest

None

### Author Contributions

MJ and RRC wrote the manuscript. MS, TK, and NLS reviewed and revised the manuscript critically. All authors approved the submission of the manuscript.

### Approval by Institutional Review Board (IRB)

Not applicable.

### Availability of Data

All the data used in this opinion paper are drawn from the references provided.
